# Conversion of methane to benzene in CVI by density functional theory study

**DOI:** 10.1038/s41598-019-56136-0

**Published:** 2019-12-20

**Authors:** Kun Li, Hejun Li, Ningning Yan, Tiyuan Wang, Wei Li, Qiang Song

**Affiliations:** State Key Laboratory of Solidification Processing, Carbon/Carbon Composites Research Center, Northwestern Polytechnical University, Xi’an, 710072 China

**Keywords:** Density functional theory, Quantum chemistry, Reaction mechanisms, Atomistic models

## Abstract

A density functional theory (DFT) study was employed to explore the mechanism of the conversion of methane to benzene in chemical vapor infiltration (CVI) based on the concluded reaction pathways from C_1_-species to C_6_-species. The geometry optimization and vibrational frequency analysis of all the chemical species and transition states (TS) were performed with B3LYP along with a basis set of 6–311 +G(d, p), and Gaussian 09 software was used to perform the study. The rate constants were calculated by KiSThelP according to the conventional transition state theory (TST), and the Wigner method was applied to acquire the tunneling correction factors. Then the rate constants were fitted to the modified Arrhenius expression in the temperature range of 800–2000 K. As for the barrierless reactions calculated in this paper, the rate constants were selected from the relating references. Through the energetic and kinetic calculations, the most favorable reaction pathway for benzene formation from methane was determined, which were mainly made of the unimolecular dissociation. The conversion trend from C_1_-species to C_4_-species is mainly guided by a strong tendency to dehydrogenation and the pathways from C_4_-species to C_6_-species are all presumed to be able to produce C_6_H_6_ molecule.

## Introduction

Chemical vapor infiltration (CVI) is a widely used method for the production of carbon/carbon (C/C) composites, which are mainly employed in the manufacture of solid rocket motor nozzles, brake discs of military and commercial aircraft, and spacecraft heat shields because of their excellent thermal and mechanical properties, like high specific modulus, high specific strength, and wear resistance at high temperature^1^. It is one of the most important ways to prepare high performance C/C composites using methane as precursor^2–10^, and the pyrolysis in the gas phase has been reported to play a significant role in the formation of C/C composites^8,11^. To fully explain the process of the gas-phase reactions and therefore help to understand and optimize the production process of C/C composites, it is essential to study the mechanism of the homogeneous reactions involved in the gas phase.

There are some researches that have been performed to explore the mechanism of the decomposition of methane. Becker and Huttinger^12^ proposed a detailed reaction scheme for pyrocarbon deposition from methane, which illustrated the reaction pathways from methane to C_6_-species and was used to simulate the pyrocarbon deposition kinetics. Huttinger^13^ presented a simplified reaction scheme for the deposition process from methane which helped to explain the deposition chemistry and kinetics. Kahle *et al*.^14^ studied the reaction pathways of the conversion of methane and concluded that coke precursor was produced from methane. Hiblot *et al*.^15^ performed an experimental and modeling study for the pyrolysis of methane and found that the methyl radical formed from methane mainly combined with another methyl radical to form C_2_-species and the H_2_ molecule could strongly limit the formation of C_2_-species. Although the above researches studied the decomposition of methane, they were unable to illustrate the nature of the mechanism of the homogeneous reactions during the pyrolysis of methane, and these results could not be explained from the view of the molecular level. Besides, the presence of the species formed during the decomposition process could not easily be detected and distinguished using the online analysis instruments, which made it quite difficult to explore the corresponding mechanisms.

Nowadays, density functional theory (DFT) has become a powerful method to study the mechanisms of the chemical reactions for its convenience and accuracy, and it has also been applied to the research of the decomposition of methane. ViCes *et al*.^16^ calculated the complete dehydrogenation of methane on platinum catalysis by DFT method and found that species like CH_3_ and CH_2_ were stabilized at edges and corners of the particles, which helped to study the surface reactivity of extended transition-metal terraces and nanoparticles. To further elucidate the reaction mechanism of the conversion of methane on the single iron sites embedded in a silica matrix, Guo *et al*.^17^ simulated the reaction profile of CH_3_ radicals with DFT and displayed a simplified reaction pathway from CH_3_ to C_6_H_6_. Huang *et al*.^18^ presented a DFT study on the sequential process of methane decomposition on iron oxides, which could provide a detailed explanation of the reaction mechanism at the atomistic for the design of more efficient oxygen carriers. Besides, in order to understand the activity of heterogeneous catalysis affected by nanostructure, Kozlov *et al*.^19^ calculated the complete methane dehydrogenation on nanostructured palladium by DFT. Despite the plenty of theoretical studies of the decomposition of methane published already, there are few DFT researches concerning the detailed conversion pathways of methane to benzene in CVI, and it is of great necessity to figure out the mechanism from the molecular level.

During the decomposition of methane in CVI, benzene is a basic element of pyrolytic aromatic hydrocarbons which can further form the pyrocarbon, thus the present work is performed by limiting the reaction route only to the first cyclic ring formation. In this work, a DFT study was employed to explore the mechanism of the conversion of methane to benzene in CVI based on the concluded reaction pathways from C_1_-species to C_6_-species. The energy profiles of each reaction were calculated and the corresponding rate constants were obtained subsequently, then the most favorable reaction pathway was proposed.

## Computational Details

All the chemical species involved in the study were optimized with different spin multiplicities followed by vibrational frequency calculations to obtain the stable molecular structures that provided the lowest energy and correct geometry. In order to verify the structure of the transition states (TS) of all the chemical reactions, the frequency analysis was conducted after the geometry optimization, which turned out that each stable TS was equipped with only one imaginary frequency. Besides, the intrinsic reaction coordinate (IRC)^20^ was performed to prove that each TS was connected to the desired reactant and product. To get the IRC results with high quality and complete curve, the max points of each IRC calculation were set to be 150 coupled with the default step size of 10. The relative energies of the species and TS were applied to figure out the corresponding reaction energies and activation energies which were used for the energy profiles. All the quantum calculations were performed with Becke three-parameter exchange and Lee, Yang and Parr correlation function (B3LYP)^21,22^ along with a basis set of 6–311 +G(d, p), and Gaussian 09^23^ software was used to study all the DFT calculations.

The rate constants as a function of temperature for the reactions involved in the study were calculated by using the kinetic and statistical thermodynamical package (KiSThelP)^24^ according to the conventional transition state theory (TST)^25^. The parameters required by TST such as molecular energy, spin multiplicity, and vibrational frequencies were obtained from the Gaussian output files. As for the quantum tunneling effect, the Wigner method^26,27^ was applied to acquire the tunneling correction factors for all the elementary reactions. Besides, the total reaction degeneracies of each case were taken into account by multiplying a symmetry number of each reaction by the number of equivalent abstractable hydrogen atoms^28^. Finally, the rate constants were fitted to the modified Arrhenius expression^26,28^ in the temperature range of 800–2000 K. The formula is expressed as:

1 $${\rm{k}}({\rm{T}})={\rm{A}}\times {T}^{n}\times \exp \,(\frac{-{E}_{a}}{RT})$$

where A is the Arrhenius prefactor, T is the temperature, E_a_ is the barrier height, and n is the temperature exponent indicating the deviation from the standard Arrhenius equation. In addition, due to the fact that KiSThelP is only programmed for the reactions with TS, and for the barrierless reactions calculated in this paper, the rate constants were selected from the relative references published before.

## Results and Discussion

**Figure 1:**
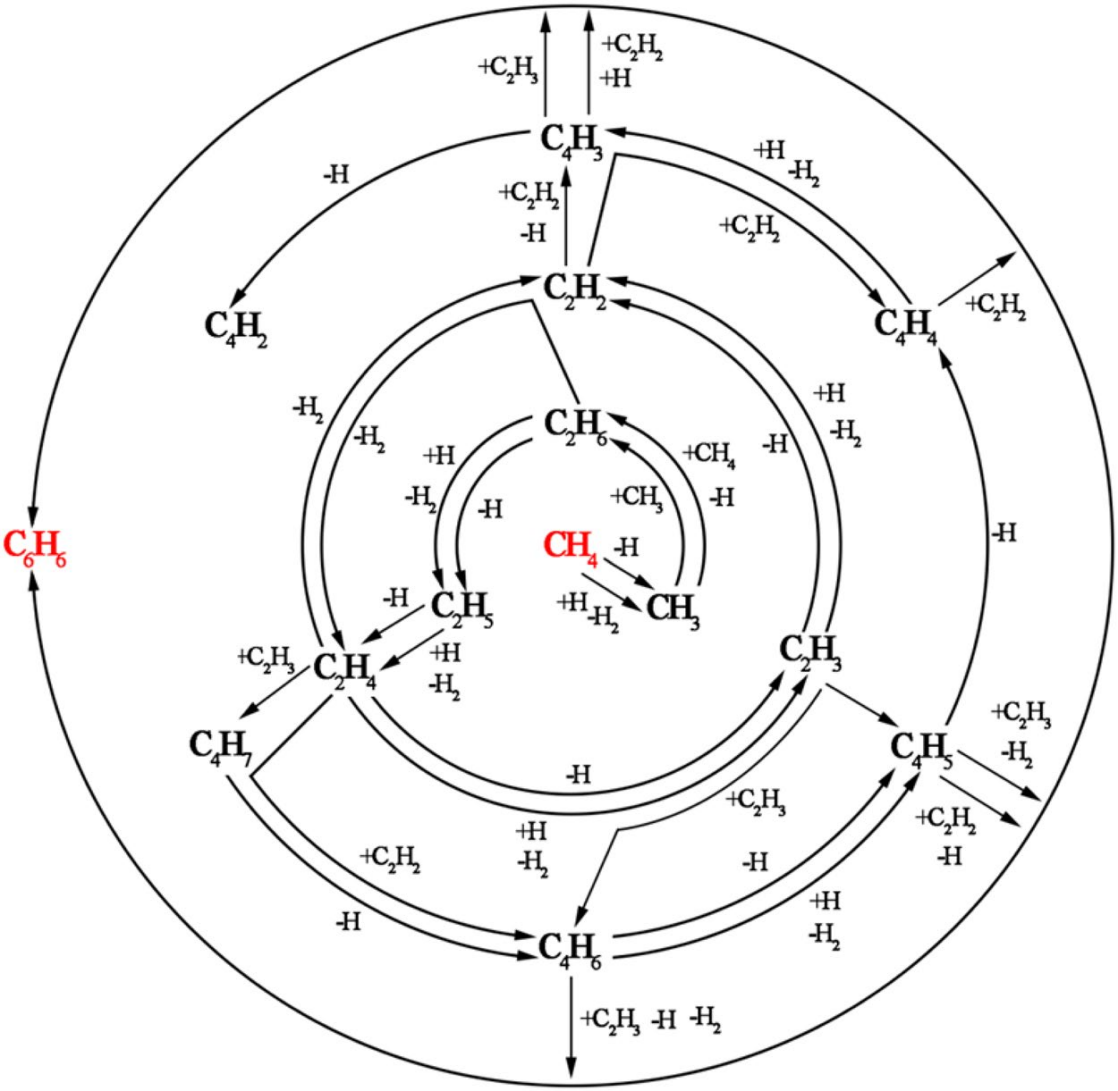
Detailed reaction scheme about the conversion way of methane to benzene.

The primary goal of this section is to obtain the main reaction pathways of the decomposition of methane during CVI by distinguishing the pathways collected from the previous researches^11,12,29–40^. By studying and summarizing the related researches published previously, detailed reaction schemes for benzene to be prepared using methane as precursor are summarized, as shown in Fig. 1. Since there are some species produced during the decomposition of methane that make little impact on the mechanism of the formation of pyrolytic carbon, it is generally accepted that only the important molecules and radicals which determine the mechanism of the pyrolysis are supposed to be focused on. As a matter of fact, it is widely agreed that the decomposition and formation of hydrocarbon are fundamentally a free-radical mechanism^11^, so most of the concluded reactions are radical ones. As depicted in Fig. 1, from inside to outside, it shows the conversion pathway from methane to C_2_-species, C_4_-species and C_6_-species, respectively.

**Figure 2:**
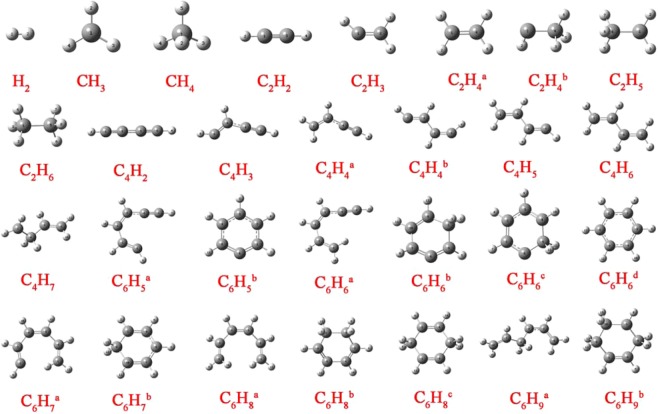
Optimized geometries of various reactants and products involved in the conversion of methane to benzene.

The molecular structures of all the reactants and products studied in this work are displayed in Fig. 2 ranging from hydrogen molecule to benzene. As can be seen from this figure, apart from the common species with only one specific structure, there are some isomers in this paper such as C_6_H_6_ that has four different chemical structures. The reaction pathways proposed in the following are all involved with the dominant species mentioned above.

### Reaction pathways from C_1_-species to C_2_-species

**Figure 3:**
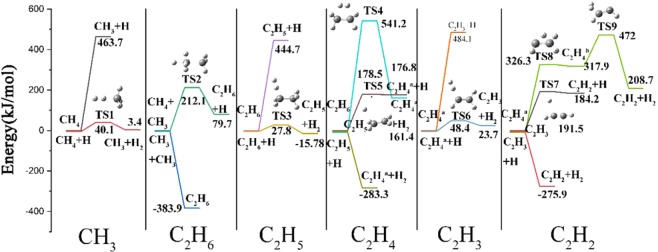
The pathways and the potential energy of the reactions from C_1_-species to C_2_-species.

For the discussion on the mechanism of reaction pathways from C_1_-species to C_2_-species, the reaction profiles containing every reaction pathway and their potential energies are displayed in Fig. 3, where the energies of each species and TS have been plotted relative to the total energy of the reactants that are all set to be 0 kJ/mol^41,42^. The reactions contributing to the same product have been divided into the same column, which are arranged from left to right in substantially the order of the formation of hydrocarbon shown in Fig. 1.

The initial step of the whole reaction system is the dehydrogenation of CH_4_ molecule, which decomposes directly into CH_3_ radical and H atom with the endoergic energy of 463.7 kJ/mol, and this is consistent with the results calculated before^17,43,44^, which proves the accuracy of this calculation method. With H atom generated, it can combine with CH_4_ molecule to produce CH_3_ radical and H_2_ molecule through TS1 with the activation energy of 40.1 kJ/mol. At the second column, two methyl can bond together to produce an ethane molecule, which is a barrierless process with the exothermic energy of 383.9 kJ/mol. The methyl can also react with methane to yield ethane and hydrogen atom by overcoming the energy barrier of 212.1 kJ/mol, and the total energy of the products is 79.7 kJ/mol higher than that of the reactants. At the column of C_2_H_5_, C_2_H_6_ molecule can be abstracted by an external H atom to form C_2_H_5_ radical and H_2_ molecule by conquering the energy barrier of 27.8 kJ/mol corresponding to TS3, and the energy of products lies 15.8 kJ/mol below the reactants. C_2_H_6_ molecule can also undergo H-elimination reaction forming C_2_H_5_ radical and H atom with the barrierless endothermic energy of 444.7 kJ/mol. As for the formation of C_2_H_4_ molecule, it can come from both C_2_H_6_ molecule and C_2_H_5_ radical. After overcoming the high energy barrier of 541.2 kJ/mol, C_2_H_4_ molecule and H_2_ molecule can be produced from C_2_H_6_ molecule with the reaction energy being 161.4 kJ/mol, indicating an endothermic reaction. C_2_H_5_ radical can decompose into C_2_H_4_ molecule and H atom by the dehydrogenation reaction, of which the activation energy and reaction energy are 178.5 kJ/mol and 176.8 kJ/mol, respectively. This column also involves the abstraction of H atom from C_2_H_5_ radical by an external H atom to form C_2_H_4_ molecule and H_2_ molecule with the barrierless reaction energy lying 283.3 kJ/mol below the reactants. The fifth column shows two endothermic reactions to the formation of C_2_H_3_ radical. By losing one H atom of C_2_H_4_ molecule, C_2_H_3_ radical can be produced with the high barrierless reaction energy being 484.1 kJ/mol. And with an extraneous H atom attacking C_2_H_4_ molecule, the corresponding products are C_2_H_3_ radical and H_2_ molecule with the energy barrier and reaction energy being 48.4 kJ/mol and 23.7 kJ/mol, respectively. Finally, the rightmost column of Fig. 3 shows the energy process of the formation of C_2_H_2_ molecule. The most complicated pathway is the formation of C_2_H_2_ molecule and H_2_ molecule from the dehydrogenation of C_2_H_4_ molecule, which gets the reaction energy of 208.7 kJ/mol after overcoming the energy barriers of 326.3 kJ/mol and 472 kJ/mol corresponding to TS8 and TS9, respectively. Another endothermic reaction is the H atom elimination of C_2_H_3_ radical, which overcomes the energy barrier of 191.5 kJ/mol and results in the energy of products being 184.2 kJ/mol. The last reaction is exothermic and barrierless, C_2_H_3_ + H → C_2_H_2_ + H_2_, and the energy of products is 275.9 kJ/mol below than that of reactants.

### Reaction pathways from C_2_-species to C_4_-species

**Figure 4:**
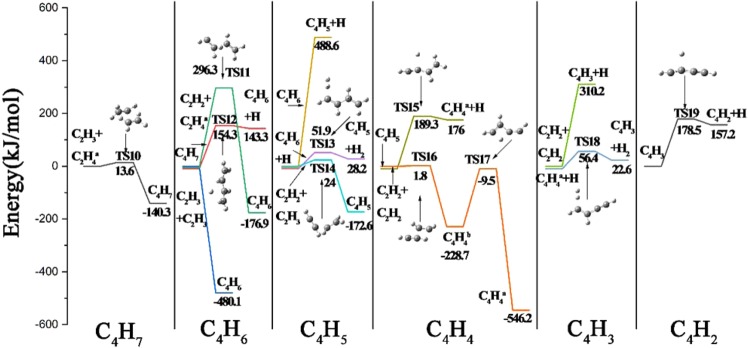
The pathways and the potential energy of the reactions from C_2_-species to C_4_-species.

The aim of this section is to deal with the different reaction pathways from C_2_-species to C_4_-species, and the corresponding potential energies of the possible and reasonable pathways are shown in Fig. 4. As is discussed in section 3.1, the same means of expression is applied to Fig. 4, which displays the formation from C_4_H_7_ radical to C_4_H_2_ molecule.

As is depicted at the first column of Fig. 4, there is only one feasible pathway leading to C_4_H_7_ radical, which comes from the combination of C_2_H_3_ radical and C_2_H_4_ molecule, and the reaction energy of this exothermic process is 140.3 kJ/mol with the energy barrier of 13.6 kJ/mol. At the second column of C_4_H_6_ molecule, three distinct reactions are calculated. The bonding of C_2_H_2_ molecule and C_2_H_4_ molecule results to the formation of C_4_H_6_ molecule with releasing 176.9 kJ/mol, after undergoing TS11 with the energy barrier of 296.3 kJ/mol. C_4_H_6_ molecule can also be formed by the dehydrogenation of C_4_H_7_ radical, which requires the activation energy of 154.3 kJ/mol, and the energy of products is 143.3 kJ/mol higher than that of reactant. Another reaction leading to the appearance of C_4_H_6_ molecule is the radical association of two C_2_H_3_ radical, which is observed to be a barrierless process with the exothermic energy being 480.1 kJ/mol. With C_4_H_6_ molecule brought into being, the C_4_H_5_ radical can be generated consequently. As displayed at the third column of Fig. 4, on the one hand, by breaking one C-H bond of C_4_H_6_ molecule, the C_4_H_5_ radical is raised with the energy of the products being 488.6 kJ/mol. On the other hand, by attacking C_4_H_6_ molecule with an external H atom, the C_4_H_5_ radical can be formed as well with another product of H_2_ molecule. This step is slightly endoergic (28.2 kJ/mol) and presents the energy barrier of 51.9 kJ/mol corresponding to TS13. Besides, C_4_H_5_ radical can also be brought out by the chemical integration of C_2_H_3_ radical and C_2_H_2_ radical, which loses 172.6 kJ/mol after conquering the energy barrier of 24 kJ/mol corresponding to TS14. At the fourth column demonstrated in Fig. 4, the formation of C_4_H_4_ molecule is analyzed by two reactions. Like the H-abstraction of C_4_H_7_ radical, C_4_H_5_ radical can decompose into C_4_H_4_ molecule and H atom by dehydrogenation, which is calculated to be endoergic (176 kJ/mol) and needs to overcome the energy barrier of 189.3 kJ/mol. Another reaction is the combination of two acetylene molecules, which goes through one intermediate of C_4_H_4_^b^ radical and two transition states of TS16 and TS17. This complicated process needs to overcome a small energy barrier of TS16 to form C_4_H_4_^b^, the energy of which is 228.7 kJ/mol lower than that of the reactants, and then C_4_H_4_^b^ radical is transformed into the more stable structure of C_4_H_4_^a^ molecule after overcoming the barrier energy of TS17. As can be observed from the structures of C_4_H_4_^a^ molecule, C_4_H_4_^b^ radical and TS17, which are illustrated in Figs. 2 and 4, C_4_H_4_^a^ molecule is transformed from C_4_H_4_^b^ radical by moving one H atom to the end of the carbon chain. At the fifth column of Fig. 4, the interaction of two acetylene molecules contributes to the presence of C_4_H_3_ radical and H atom, which is calculated to be barrierless and endothermic. Another endothermic reaction is the interaction of C_4_H_4_^a^ molecule and H forming C_4_H_3_ radical and H_2_ molecule with the energy of 22.6 kJ/mol, which undergoes TS18 of 56.4 kJ/mol. The last part of this diagram is the formation of C_4_H_2_ molecule, which is produced by abstracting the H atom of C_4_H_3_ radical, as can be seen from the structure of TS19. The energies of TS19 and the products are 178.5 kJ/mol and 157.2 kJ/mol, respectively.

### Reaction pathways from C_4_-species to C_6_-species

**Figure 5:**
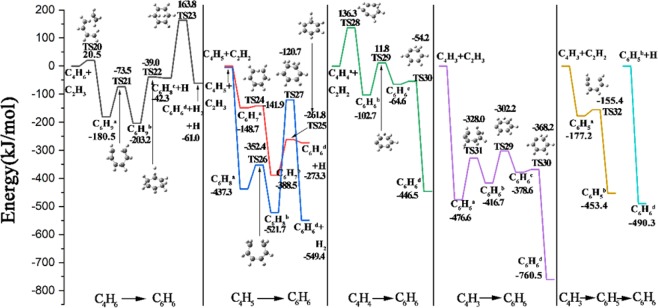
The pathways and the potential energy of the reactions from C_4_-species to C_6_-species.

This part discusses the reaction pathways leading to benzene, which is originated from C_4_H_6_ molecule, C_4_H_5_ radical, C_4_H_4_ molecule, and C_4_H_3_ radical, respectively. All the reactions analyzed in the following are multistep which are more complicated than the reactions from C_1_-species to C_4_-species, and they are divided into five columns for the convenience of discussion, as shown in Fig. 5. Unlike the other two figures discussed above, due to the fact that all these series of reactions result in the same product, benzene, Fig. 5 puts the reactions with the same main reactants into one column, such as the second column of C_4_H_5_ → C_6_H_6_.

As depicted in the left column of Fig. 5, there is just one reasonable pathway resulting in benzene from C_4_H_6_ molecule, and four transition structures can be found. This process is initiated by the chemical combination of C_4_H_6_ molecule and C_2_H_3_ radical that brings C_6_H_9_^a^ radical into existence after overcoming the small energy barrier of 20.5 kJ/mol. Then C_6_H_9_^a^ radical undergoes a ring closure reaction after overcoming the activation energy of TS21 to form C_6_H_9_^b^ molecule, which lies 203.2 kJ/mol below the reactants. And for the reaction to proceed further, benzene can arise from the dehydrogenation of C_6_H_9_^b^ molecule followed by subsequent decomposition of C_6_H_8_^c^ molecule into C_6_H_6_ molecule and H_2_ molecule, which goes through two TS configurations of TS22 and TS23. At the second column, the interactions between C_4_H_5_ radical and C_2_H_2_ molecule or C_2_H_3_ radical are calculated separately. The addition of C_2_H_2_ molecule takes place on C_4_H_5_ radical to form C_6_H_7_^a^ radical, which turns out to be a barrierless and exothermic process with the energy below than that of the reactants by 148.7 kJ/mol. Then C_6_H_7_^a^ radical undergoes a ring closure step after overcoming a small energy barrier of TS24 to produce a more stable structure of C_6_H_7_^b^ radical, which lies 388.5 kJ/mol below the group of C_4_H_5_ radical and C_2_H_2_ molecule. After that, the C-H bond scission happens to C_6_H_7_^b^ radical to generate benzene molecule and H atom, and TS25 is observed during this endothermic step with the energy of the final products being 273.3 kJ/mol lower when compared to the initial reactants. As for the addition of C_2_H_3_ radical to C_4_H_5_ radical, the whole process is very similar to the addition from C_2_H_2_ radical to C_4_H_5_ radical. At first, C_6_H_8_^a^ molecule is produced with its energy lower than the reactants by 437.3 kJ/mol after the chemical integration of C_2_H_3_ radical and C_4_H_5_ radical, which is proved to be barrierless and exothermic. And C_6_H_8_^a^ molecule is then transformed into C_6_H_8_^b^ molecule by the ring closure step, which needs to conquer the energy barrier of TS26 and release energy to achieve the stable structure of C_6_H_8_^b^ molecule with its energy lying 521.7 kJ/mol below the reactants. Further, in the third step of this route, two neighboring hydrogen atoms of C_6_H_8_^b^ molecule ruptured from C−H bonds tend to form a dihydrogen molecule leaving a stable benzene molecule, which needs to overcome the high activation energy of TS27.

As displayed in the middle column of Fig. 5, the coupling of C_4_H_4_ molecule and C_2_H_2_ molecule resulting in benzene is calculated, which undergoes three transition structures TS28, TS29 and TS30, respectively. The first step of this route is exothermic (−102.7 kJ/mol) and presents the activation energy of 136.3 kJ/mol, which brings the C_6_H_6_^b^ species out. Then there are two steps of H atom migration on C_6_H_6_^b^ species leading to benzene molecule eventually after overcoming the energy barriers of TS29 and TS30, respectively. And the energy of the benzene molecule is 446.5 kJ/mol lower than that of the beginning reactants showing the exothermicity of this process. As for the fourth column of Fig. 5, at first glance, the chemical mechanism for this route seems as complicated as that described for the conversion from C_4_H_4_ molecule to benzene molecule. It begins with the addition of C_2_H_3_ radical to C_4_H_3_ radical forming C_6_H_6_^a^ species by releasing 476.6 kJ/mol without overcoming the energy barrier. Then C_6_H_6_^a^ species is converted to C_6_H_6_^b^ species by conquering the activation energy of TS31. From the next step on, the following process is exactly the same as the conversion from C_6_H_6_^b^ species to benzene molecule described in the third column except for the absolute values marked in Fig. 5. Finally, in the last column of Fig. 5, it demonstrates the conversion from C_4_H_3_ radical to the benzene molecule. Actually, this process is composed of two separate reactions, which is connected by the C_6_H_5_^b^ radical. At first, the chemical bonding of species C_4_H_3_ radical and C_2_H_2_ molecule takes place resulting in the presence of C_6_H_5_^a^ radical, which releases 177.2 kJ/mol without any activation energy. Then C_6_H_5_^a^ radical is converted to a phenyl radical after overcoming the slight energy barrier of TS32. Due to the instability of phenyl radical, it is very possible for it to bond with an external H atom to form benzene molecule, which is calculated to be barrierless and releases the energy of 490.3 kJ/mol. Because the external H atom is added during this process rather than in the beginning, it makes sense to draw the energy profiles of these two reactions separately to illustrate this process more clearly and correctly.

### Reaction kinetics

To figure out the reactions among those discussed above that play important roles during the conversion of methane to benzene, reaction kinetics of the elementary reactions were calculated and used for kinetic simulations, as illustrated below.

Table 1 provides the kinetic parameters for elementary reactions with TS among the reactions of C_1_–C_6_ in the temperature range of 800–2000 K which covers the possible formation temperature of the species studied in this work, and for the benefit of the analysis of the mechanisms of these reactions the rate constants of the corresponding reactions in the temperature of 1353 K are listed individually because this temperature is very special during the decomposition of methane by using CVI method^9^. As is shown in Table 1, the kinetic parameters for the 32 reactions differ greatly with each other with the difference of the class and progress of the reactions. For example, the lowest reaction rate in 1353 K is 4.30 × 10^−22^ cm^−3^ mol^−1^ s^−1^ of C_2_H_2_ + C_2_H_4_ → C_4_H_6_, and the highest reaction rate in 1353 K is 1.52 × 10^12^ cm^−3^ mol^−1^ s^−1^ of C_6_H_6_^c^ → C_6_H_6_^d^, which shows a difference of 34 orders of magnitude between these two reaction rates.

**Table 1:** Kinetic parameters for elementary reactions with transition states among the reactions of C_1_–C_6_.

Reaction	A	n	E_a_	k (T = 1353 K)
C_1_–C_2_				
CH_4_ + H → CH_3_ + H_2_	8.20 × 10^−17^	2.01	27.0	1.46 × 10^−11^
CH_3_ + CH_4_ → C_2_H_6_ + H	5.28 × 10^−24^	3.52	197.3	1.38 × 10^−20^
C_2_H_6_ + H → C_2_H_5_ + H_2_	1.04 × 10^−16^	2.04	13.7	7.45 × 10^−11^
C_2_H_6_ → C_2_H_4_ + H_2_	5.08 × 10^10^	1.19	509.6	5.82 × 10^−6^
C_2_H_5_ → C_2_H_4_ + H	4.56 × 10^11^	0.61	163.7	1.78 × 10^7^
C_2_H_4_ + H → C_2_H_3_ + H_2_	2.25 × 10^−16^	1.95	36.2	1.17 × 10^−11^
C_2_H_3_ → C_2_H_2_ + H	1.58 × 10^12^	0.69	175.9	3.68 × 10^7^
C_2_H_4_^a^ → C_2_H_4_^b^	2.41 × 10^12^	0.49	312.3	7.13 × 10^1^
C_2_H_4_^b^ → C_2_H_2_ + H_2_	2.99 × 10^11^	0.61	139.7	1.01 × 10^8^
C_2_–C_4_				
C_2_H_3_ + C_2_H_4_ → C_4_H_7_	1.75 × 10^−21^	3.02	14.4	1.38 × 10^−12^
C_2_H_2_ + C_2_H_4_ → C_4_H_6_	3.84 × 10^−22^	3.23	260.9	4.30 × 10^−22^
C_4_H_7_ → C_4_H_6_ + H	1.23 × 10^11^	0.64	138.7	5.45 × 10^7^
C_4_H_6_ + H → C_4_H_5_ + H_2_	8.23 × 10^−17^	1.91	41.0	2.10 × 10^−12^
C_2_H_2_ + C_2_H_3_ → C_4_H_5_	2.43 × 10^−19^	2.52	−41.7	7.68 × 10^−10^
C_4_H_5_ → C_4_H_4_^a^ + H	9.62 × 10^11^	0.67	160.6	7.61 × 10^7^
C_2_H_2_ + C_2_H_2_ → C_4_H_4_^b^	2.26 × 10^−16^	2.24	232.1	2.48 × 10^−18^
C_4_H_4_^b^ → C_4_H_4_^a^	1.33 × 10^11^	0.65	201.1	2.53 × 10^5^
C_4_H_4_^a^ + H → C_4_H_3_ + H_2_	1.08 × 10^−16^	1.91	45.5	1.76 × 10^−12^
C_4_H_3_ → C_4_H_2_ + H	7.05 × 10^11^	0.71	161.5	7.04 × 10^7^
C_4_–C_6_				
C_4_H_6_ + C_2_H_3_ → C_6_H_9_^a^	4.24 × 10^−22^	3.01	19.7	1.94 × 10^−13^
C_6_H_9_^a^ → C_6_H_9_^b^	9.20 × 10^10^	0.11	106.6	1.51 × 10^7^
C_6_H_9_^b^ → C_6_H_8_^c^ + H	7.60 × 10^11^	0.64	149.3	1.30 × 10^8^
C_6_H_8_^c^ → C_6_H_6_^d^ + H_2_	1.67 × 10^9^	1.00	180.7	2.47 × 10^5^
C_6_H_7_^a^ → C_6_H_7_^b^	6.70 × 10^11^	0.06	5.6	6.23 × 10^11^
C_6_H_7_^b^ → C_6_H_6_^d^ + H	4.68 × 10^11^	0.60	112.5	1.55 × 10^9^
C_6_H_8_^a^ → C_6_H_8_^b^	1.35 × 10^11^	0.09	82.9	1.66 × 10^8^
C_6_H_8_^b^ → C_6_H_6_^d^ + H_2_	5.65 × 10^9^	1.20	379.1	7.42 × 10^−2^
C_4_H_4_^a^ + C_2_H_2_ → C_6_H_6_^b^	2.13 × 10^−22^	2.62	67.4	8.45 × 10^−17^
C_6_H_6_^b^ → C_6_H_6_^c^	6.11 × 10^11^	0.35	105.2	6.71 × 10^8^
C_6_H_6_^c^ → C_6_H_6_^d^	8.71 × 10^11^	0.16	6.4	1.52 × 10^12^
C_6_H_6_^a^ → C_6_H_6_^b^	6.26 × 10^10^	0.16	144.9	5.06 × 10^5^
C_6_H_5_^a^ → C_6_H_5_^b^	1.02 × 10^12^	−0.02	21.0	1.37 × 10^11^

Rate constants are fitted in the modified Arrhenius form (k = AT^n^e^−Ea/RT^) in the temperature range of 800–2000 K. Values of the parameters A and E_a_ are in units of cm^3^ s^−1^ molecule^−1^ and kJ mol^−1^, respectively. Values of k (T) are reported at 1353 K.

Table 2 displays the reaction rates for 13 elementary reactions without TS among the reactions of C_1_–C_6_. Because the KiSThelP is unable to calculate the rate constants of the barrierless reactions, the corresponding rate values are taken from the literatures by searching the NIST chemical kinetics database^45^. To the best of our knowledge, there are no relevant theoretical or experimental results about the rate constants for the reaction C_4_H_3_ + C_2_H_3_ → C_6_H_6_^a^, and as a result, this specific reaction is disappeared in Table 2. As can be seen, almost all the rate constants are selected at the temperature of 1400 K which is close to 1353 K and can be valuable as well, except for the reaction C_2_H_3_ + C_2_H_3_ → C_4_H_6_ whose rate constant is elected at 298 K because of the lack of its kinetic study at high temperatures. Like Table 1, the rate constants of these barrierless reactions dispread in a wide range. The smallest value is 5.00 × 10^−21^ cm^−3^mol^−1^s^−1^ of reaction CH_4_ → CH_3_ + H, and the largest value is 13.70 cm^−3^mol^−1^s^−1^ of reaction C_2_H_6_ → C_2_H_5_ + H, which indicates that all the rate constants of the barrierless reactions are commonly low.

**Table 2:** Kinetic parameters for elementary reactions without transition states among the reactions of C_1_–C_6_. Values of k (T) are taken from the literatures.

Reaction	k (T)	References
C_1_–C_2_		
CH_4_ → CH_3_ + H	k (1400 K) = 5.00 × 10^−21^	^46^
CH_3_ + CH_3_ → C_2_H_6_	k (1400 K) = 3.04 × 10^−11^	^47^
C_2_H_6_ → C_2_H_5_ + H	k (1400 K) = 13.70	^48^
C_2_H_5_ + H → C_2_H_4_^a^ + H_2_	k (1400 K) = 3.01 × 10^−12^	^49^
C_2_H_4_^a^ → C_2_H_3_ + H	k (1400 K) = 0.13	^50^
C_2_H_3_ + H → C_2_H_2_ + H_2_	k (1400 K) = 2.01 × 10^−11^	^51^
C_2_–C_4_		
C_2_H_3_ + C_2_H_3_ → C_4_H_6_	k (298 K) = 9.50 × 10^−11^	^52^
C_4_H_6_ → C_4_H_5_ + H	k (1400 K) = 1.04	^53^
C_2_H_2_ + C_2_H_2_ → C_4_H_3_ + H	k (1200 K) = 1.60 × 10^−24^	^54^
C_4_–C_6_		
C_2_H_2_ + C_4_H_5_ → C_6_H_7_^a^	k (1400 K) = 1.31 × 10^−14^	^55^
C_2_H_3_ + C_4_H_5_ → C_6_H_8_^a^	k (1400 K) = 1.39 × 10^−11^	^55^
C_4_H_3_ + C_2_H_2_ → C_6_H_5_^a^	k (1400 K) = 3.20 × 10^−14^	^55^
C_6_H_5_ + H → C_6_H_6_^d^	k (1400 K) = 2.05 × 10^−10^	^56^

**Figure 6:**
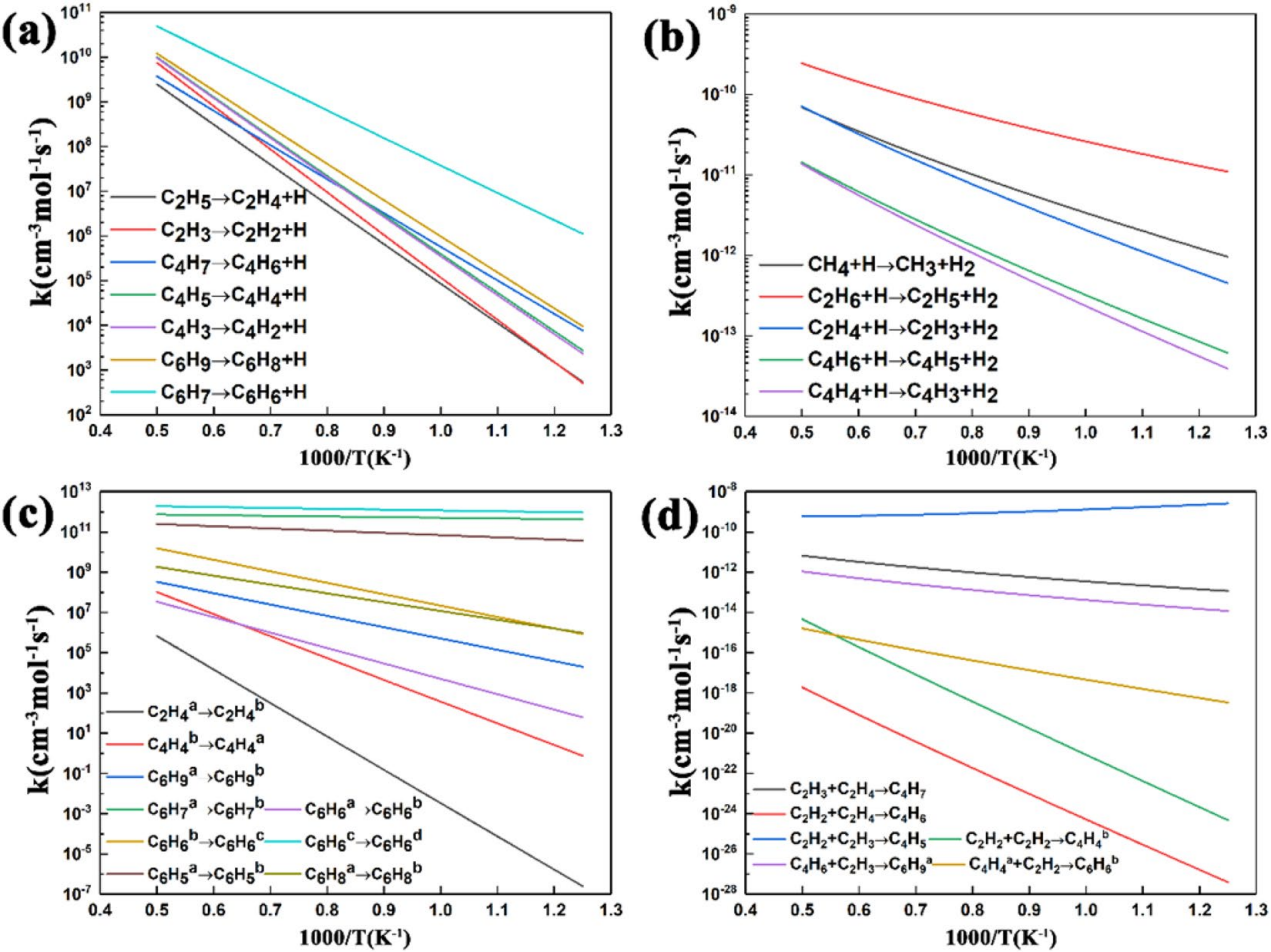
Comparisons of calculated rate constants for (**a**) dehydrogenation, (**b**) H-abstraction by H atom, (**c**) isomerization and (**d**) combination among the reactions of C_1_–C_6_.

To better understand the variation tendency of the rate constants of the reactions listed in Table 1, Fig. 6 presents a comparison of those reactions, which are divided into four parts due to the different reaction classes, dehydrogenation, H-abstraction by H atom, isomerization and combination. As demonstrated in Fig. 6, through the detailed comparisons of the rate constants within the same reaction class, it is concluded that the ranges of the rate constants for dehydrogenation reactions and H-abstraction reactions are both relatively small in the temperature range of 800–2000 K when compared with the isomerization reactions and combination reactions whose rate constants vary greatly with the increase of temperature. The reason may be due to that the rate constants for similar reactions are mainly dependent on the molecular structure. In general, the rate constants of all those reactions increase with the increase of temperature, except for the reaction C_2_H_2_ + C_2_H_3_ → C_4_H_5_ whose rate constants decrease gently with the increase of temperature, illustrated in Fig. 6d. Further, there are a few reactions showing high rate constants all the time, such as the reaction C_6_H_6_^c^ → C_6_H_6_^d^ in Fig. 6c, which is attributed to the fact that the less stable radical has changed into the reasonably stabilized molecule with low energy. In addition, there are another 4 reactions that are not included in Fig. 6, C_2_H_6_ → C_2_H_4_ + H_2_, C_2_H_4_^b^ → C_2_H_2_ + H_2_, C_6_H_8_^b^ → C_6_H_6_^d^ + H_2_ and C_6_H_8_^c^ → C_6_H_6_^d^ + H_2_. Obviously, the dissociation of these 4 reactions is quite different from the previous four reaction classes, and the difference of the orders of magnitude among these four reaction rates are too large to be plotted in one figure. However, the rate constants of these reactions still increase with the increase of temperature.

## General Discussion

**Figure 7:**
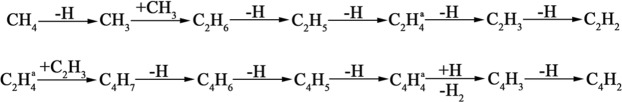
Main reaction route from C_1_ to C_4_ during the conversion of methane to benzene.

It is widely accepted that all the reactions proposed above are possible pathways for the overall process of the conversion of methane to benzene in CVI. Furthermore, based on the above analysis, the most preferred pathway can be identified by comparing the potential energies and rate constants of the reactions resulting in the same products, concluded in Table 3. On the one hand, the exothermic reactions are more preferred than the endothermic reactions. For example, for the production of C_2_H_6_ molecule in Fig. 3, the reaction CH_3_ + CH_3_ → C_2_H_6_ has an advantage over the reaction CH_3_ + CH_4_ → C_2_H_6_ + H because of its exothermicity. On the other hand, the reactions with higher rate constants are more favorable. For instance, between the two reactions forming C_2_H_5_ radical in Fig. 3, the reaction C_2_H_6_ → C_2_H_5_ + H is preferential rather than the reaction C_2_H_6_ + H → C_2_H_5_ + H_2_ because the rate constant of the former reaction is 11 orders of magnitude higher than that of the latter one. And by adopting the two rules into all the reactions, the concluded main pathway is drawn in Fig. 7. Meanwhile, the high energy inputs required for some of the reactions are provided by the high-temperature environment. As for the pathways from C_4_-species to C_6_-species, each reaction route is composed of at least three steps and exhibits similar potential energies and rate constants, which makes it quite difficult to distinguish the most favorable route among these reactions. Consequently, it is inferred that C_6_H_6_ molecule can be possibly produced through all these routes shown in Fig. 5.

**Table 3:** Comparisons of the potential energies and rate constants of the reactions.

Product	Reactions	Type	k	Main reaction
CH_3_	CH_4_ + H → CH_3_ + H_2_	exothermic	1.46 × 10^−11^	CH_4_ → CH_3_ + H
	CH_4_ → CH_3_ + H	endothermic	5.00 × 10^−21^	
C_2_H_6_	CH_3_ + CH_4_ → C_2_H_6_ + H	endothermic	1.38 × 10^−20^	CH_3_ + CH_3_ → C_2_H_6_
	CH_3_ + CH_3_ → C_2_H_6_	exothermic	3.04 × 10^−11^	
C_2_H_5_	C_2_H_6_ + H → C_2_H_5_ + H_2_	exothermic	7.45 × 10^−11^	C_2_H_6_ → C_2_H_5_ + H
	C_2_H_6_ → C_2_H_5_ + H	endothermic	13.70	
C_2_H_4_	C_2_H_6_ → C_2_H_4_ + H_2_	endothermic	5.82 × 10^−6^	C_2_H_5_ → C_2_H_4_ + H
	C_2_H_5_ → C_2_H_4_ + H	endothermic	1.78 × 10^7^	
	C_2_H_5_ + H → C_2_H_4_^a^ + H_2_	exothermic	3.01 × 10^−12^	
C_2_H_3_	C_2_H_4_ + H → C_2_H_3_ + H_2_	endothermic	1.17 × 10^−11^	C_2_H_4_^a^ → C_2_H_3_ + H
	C_2_H_4_^a^ → C_2_H_3_ + H	endothermic	0.13	
C_2_H_2_	C_2_H_3_ → C_2_H_2_ + H	endothermic	3.68 × 10^7^	C_2_H_3_ → C_2_H_2_ + H
	C_2_H_3_ + H → C_2_H_2_ + H_2_	exothermic	2.01 × 10^−11^	
C_4_H_7_	C_2_H_3_ + C_2_H_4_ → C_4_H_7_	exothermic	1.38 × 10^−12^	C_2_H_3_ + C_2_H_4_ → C_4_H_7_
C_4_H_6_	C_2_H_2_ + C_2_H_4_ → C_4_H_6_	exothermic	4.30 × 10^−22^	C_4_H_7_ → C_4_H_6_ + H
	C_4_H_7_ → C_4_H_6_ + H	endothermic	5.45 × 10^7^	
	C_2_H_3_ + C_2_H_3_ → C_4_H_6_	exothermic	9.50 × 10^−11^	
C_4_H_5_	C_4_H_6_ + H → C_4_H_5_ + H_2_	endothermic	2.10 × 10^−12^	C_4_H_6_ → C_4_H_5_ + H
	C_2_H_2_ + C_2_H_3_ → C_4_H_5_	exothermic	7.68 × 10^−10^	
	C_4_H_6_ → C_4_H_5_ + H	endothermic	1.04	
C_4_H_4_	C_4_H_5_ → C_4_H_4_^a^ + H	endothermic	7.61 × 10^7^	C_4_H_5_ → C_4_H_4_^a^ + H
C_4_H_3_	C_4_H_4_^a^ + H → C_4_H_3_ + H_2_	endothermic	1.76 × 10^−12^	C_4_H_4_^a^ + H → C_4_H_3_ + H_2_
	C_2_H_2_ + C_2_H_2_ → C_4_H_3_ + H	endothermic	1.60 × 10^−24^	
C_4_H_2_	C_4_H_3_ → C_4_H_2_ + H	endothermic	7.04 × 10^7^	C_4_H_3_ → C_4_H_2_ + H

As depicted in Fig. 7, the most fundamental and initial step of this route is the dehydrogenation of CH_4_ molecule which is the original material of the process, even though this step takes a lot of energy to proceed and has a low rate constant. Besides, the dehydrogenation effect plays a significant role in the whole process. The C_2_H_4_ molecule, C_2_H_3_ radical, and C_2_H_2_ molecule produced during the dehydrogenation can help the reactions of C_4_-species to perform further, and C_2_H_3_ radical and C_2_H_2_ molecule can also take part in the production of C_6_H_6_ molecule. The multistep reactions forming C_6_H_6_ molecule are mainly attributed to the participation of C_4_H_3_ radical, C_4_H_4_ molecule, C_4_H_5_ radical, and C_4_H_6_ molecule. From Fig. 7, it can be seen that the main reaction routes are made of the unimolecular dissociation.

## Conclusions

Based on the concluded pathways from methane to benzene, the DFT study was performed to explain the reaction mechanisms and corresponding kinetics, which presented the calculation results of the potential energy and Arrhenius parameters involved in the process. The reaction profiles and rate constants of each pathway from C_1_-species to C_6_-species were obtained and the most favorable route was proposed. The conversion trend from C_1_-species to C_4_-species is mainly guided by a strong tendency to dehydrogenation, which is found to play an important role in this route. And the main reaction routes are made of the unimolecular dissociation. In addition, the pathways from C_4_-species to C_6_-species are all presumed to be able to produce C_6_H_6_ molecule.

## References

[1] Park, S. J. Carbon/Carbon Composites. In *Carbon Fibers* (ed. Park, S. J.) 279–294 (Springer 2018).

[2] Li H (2013). Anti-oxidation and ablation properties of carbon/carbon composites infiltrated by hafnium boride. Carbon..

[3] Song Q (2017). Carbon nanotube-multilayered graphene edge plane core-shell hybrid foams for ultrahigh-performance electromagnetic-interference shielding. Adv Mater..

[4] Cheng C (2018). Effects of pyrocarbon on morphology stability of SiC nanowires at high temperatures. J Am Ceram Soc..

[5] Chen M (2018). High temperature oxidation resistance of La_2_O_3_-modified ZrB_2_-SiC coating for SiC-coated carbon/carbon composites. J Alloy Compd..

[6] Hu ZJ, Zhang WG, Hüttinger KJ, Reznik B, Gerthsen D (2003). Influence of pressure, temperature and surface area/volume ratio on the texture of pyrolytic carbon deposited from methane. Carbon..

[7] Sun C, Li H, Fu Q, Zhang J (2014). Microstructure and ablation properties of carbon/carbon composites modified by ZrSiO_4_. Corros Sci..

[8] Li A, Norinaga K, Zhang W, Deutschmann O (2008). Modeling and simulation of materials synthesis: Chemical vapor deposition and infiltration of pyrolytic carbon. Compos Sci Technol..

[9] Shen Q (2018). Simultaneously improving the mechanical strength and electromagnetic interference shielding of carbon/carbon composites by electrophoretic deposition of SiC nanowires. J Mater Chem C..

[10] Song Q (2018). Vertically grown edge-rich graphene nanosheets for spatial control of Li nucleation. Adv Energy Mater..

[11] Féron O, Langlais F, Naslain R (1999). Analysis of the gas phase by *in situ* FTIR spectrometry and mass spectrometry during the CVD of pyrocarbon from propane. Chem Vapor Depos..

[12] Becker A, Hüttinger KJ (1998). Chemistry and kinetics of chemical vapor deposition of pyrocarbon — IV Pyrocarbon deposition from methane in the low temperature regime. Carbon..

[13] Hüttinger KJ (1998). CVD in hot wall reactors — The interaction between homogeneous gas-phase and heterogeneous surface reactions. Chem Vapor Depos..

[14] Kahle LCS (2013). Methane dry reforming at high temperature and elevated pressure: Impact of gas-phase reactions. Ind Eng Chem Res..

[15] Hiblot H, Ziegler-Devin I, Fournet R, Glaude PA (2016). Steam reforming of methane in a synthesis gas from biomass gasification. Int J Hydrogen Energ..

[16] Viñes F (2010). Methane activation by platinum: Critical role of edge and corner sites of metal nanoparticles. Chemistry - A European Journal..

[17] Guo X (2014). Direct, nonoxidative conversion of methane to ethylene, aromatics, and hydrogen. Science..

[18] Huang L, Tang M, Fan M, Cheng H (2015). Density functional theory study on the reaction between hematite and methane during chemical looping process. Appl Energ..

[19] Kozlov SM, Neyman KM (2016). Insights from methane decomposition on nanostructured palladium. J Catal..

[20] Gonzalez C, Schlegel HB (1990). Reaction path following in mass-weighted internal coordinates. J. Phys. Chem..

[21] Stephens PJ (1994). Ab initio calculation of vibrational absorption and circular dichroism spectra using density functional force fields. Journal of Physical Chemistry..

[22] Becke AD (1993). Density‐functional thermochemistry. III. The role of exact exchange. The Journal of Chemical Physics..

[23] Gaussian 09, Revision D.01, M. J. Frisch *et al*., Gaussian, Inc., Wallingford CT, (2013).

[24] Canneaux S, Bohr F, Henon E (2014). KiSThelP: A program to predict thermodynamic properties and rate constants from quantum chemistry results. Journal of Computational Chemistry..

[25] Truhlar DG, Garrett BC, Klippenstein SJ (1996). Current status of transition-state theory. The Journal of Physical Chemistry..

[26] Georganta E, Rahman RK, Raj A, Sinha S (2017). Growth of polycyclic aromatic hydrocarbons (PAHs) by methyl radicals: Pyrene formation from phenanthrene. Combust Flame..

[27] Raj A (2010). New polycyclic aromatic hydrocarbon (PAH) surface processes to improve the model prediction of the composition of combustion-generated PAHs and soot. Carbon..

[28] Fernández-Ramos A, Ellingson BA, Meana-Pañeda R, Marques JMC, Truhlar DG (2007). Symmetry numbers and chemical reaction rates. Theor Chem Acc..

[29] Hu C, Li H, Zhang S, Li W (2016). A molecular-level analysis of gas-phase reactions in chemical vapor deposition of carbon from methane using a detailed kinetic model. J Mater Sci..

[30] Becker A, Hüttinger KJ (1998). Chemistry and kinetics of chemical vapor deposition of pyrocarbon — III Pyrocarbon deposition from propylene and benzene in the low temperature regime. Carbon..

[31] Becker A, Hüttinger KJ (1998). Chemistry and kinetics of chemical vapor deposition of pyrocarbon—II Pyrocarbon deposition from ethylene, acetylene and 1, 3-butadiene in the low temperature regime. Carbon..

[32] Benzinger W, Becker A, Hüttinger KJ (1996). Chemistry and kinetics of chemical vapour deposition of pyrocarbon: I. Fundamentals of kinetics and chemical reaction engineering. Carbon..

[33] Antes J, Hu Z, Zhang W, Hüttinger KJ (1999). Chemistry and kinetics of chemical vapour deposition of pyrocarbon: VII Confirmation of the influence of the substrate surface area/reactor volume ratio. Carbon..

[34] Norinaga K, Deutschmann O (2007). Detailed kinetic modeling of gas-phase reactions in the chemical vapor deposition of carbon from light hydrocarbons. Ind Eng Chem Res..

[35] Frenklach M, Wang H (1991). Detailed surface and gas-phase chemical kinetics of diamond deposition. Physical review B..

[36] Sabbe M (2011). First principle-based simulation of ethane steam cracking. Aiche Journal..

[37] Ge Y, Gordon MS, Battaglia F, Fox RO (2010). Theoretical study of the pyrolysis of methyltrichlorosilane in the gas phase. 3. Reaction rate constant calculations. The Journal of Physical Chemistry A..

[38] Ziegler I, Fournet R, Marquaire PM (2005). Pyrolysis of propane for CVI of pyrocarbon: Part I. Experimental and modeling study of the formation of toluene and aliphatic species. J. Anal. Appl. Pyrolysis..

[39] Ziegler I, Fournet R, Marquaire PM (2005). Pyrolysis of propane for CVI of pyrocarbon: Part II. Experimental and modeling study of polyaromatic species. J. Anal. Appl. Pyrolysis..

[40] Ziegler I, Fournet R, Marquaire PM (2007). Pyrolysis of propane for CVI of pyrocarbon: Part III: Experimental and modeling study of the formation of pyrocarbon. J. Anal. Appl. Pyrolysis..

[41] Ma Y, Xue W, Wang Z, Ge M, He S (2008). Acetylene cyclotrimerization catalyzed by TiO_2_ and VO_2_ in the gas phase: A DFT study. The Journal of Physical Chemistry A..

[42] Huang X, Cheng D, Chen F, Zhan X (2012). The decomposition of aromatic hydrocarbons during coal pyrolysis in hydrogen plasma: A density functional theory study. Int J Hydrogen Energ..

[43] Blanksby SJ, Ellison GB (2003). Bond dissociation energies of organic molecules. Accounts Chem Res..

[44] Holmen A (2009). Direct conversion of methane to fuels and chemicals. Catal Today..

[45] Manion, J. A. *et al*. NIST Chemical Kinetics Database, NIST Standard Reference Database 17, Version 7.0 (Web Version), Release 1.6.8, Data version 2015.09, National Institute of Standards and Technology, Gaithersburg, Maryland, 20899–8320, http://kinetics.nist.gov/.

[46] Cobos CJ, Troe J (1990). The dissociation-recombination system CH_4_ + M ⇌ CH_3_ + H + M: Reevaluated experiments from 300 to 3000 K. Zeitschrift für Physikalische Chemie..

[47] Klippenstein SJ, Georgievskii Y, Harding LB (2006). Predictive theory for the combination kinetics of two alkyl radicals. Physical Chemistry Chemical Physics..

[48] Stewart PH, Larson CW, Golden DM (1989). Pressure and temperature dependence of reactions proceeding via a bound complex. 2. Application to 2CH_3_ → C_2_H_5_ + H. Combustion & Flame..

[49] Tsang W, Hampson RF (1986). Chemical kinetic data base for combustion chemistry. Part I. Methane and related compounds. Journal of Physical and Chemical Reference Data..

[50] Dean AM (1985). Predictions of pressure and temperature effects upon radical addition and recombination reactions. Journal of Physical Chemistry..

[51] Baulch DL (1992). Evaluated kinetic data for combustion modelling. J. Phys. Chem. Ref. Data..

[52] Laufer AH, Fahr A (2004). Reactions and kinetics of unsaturated C2 Hydrocarbon Radicals. Chemical Reviews..

[53] Hidaka Y (1993). Thermal isomerization and decomposition of 2-butyne in shock waves. The Journal of Physical Chemistry..

[54] Benson SW (1989). The mechanism of the reversible reaction: 2C_2_H_2_⇌ vinyl acetylene and the pyrolysis of butadiene. International Journal of Chemical Kinetics..

[55] Westmoreland PR (1989). Forming benzene in flames by chemically activated isomerization. The Journal of Physical Chemistry B..

[56] Harding LB, Georgievskii Y, Klippenstein SJ (2005). Predictive theory for hydrogen atom? Hydrocarbon radical association kinetics. The Journal of Physical Chemistry A..

